# Complete Genome Sequences of Four Brucella suis Strains Isolated from Swiss Wild Boars

**DOI:** 10.1128/MRA.01048-20

**Published:** 2020-12-03

**Authors:** Hatice Akarsu, Isabelle Brodard, Sonja Kittl, Gudrun Overesch, Joerg Jores

**Affiliations:** a Institute of Veterinary Bacteriology, University of Bern, Bern, Switzerland; University of Southern California

## Abstract

We present the complete genomes of four Brucella suis biovar 2 isolates that were obtained from wild boars in Switzerland in 2008 and 2009. Genomes were sequenced with PacBio technology, contained two chromosomes each, had a genome size of 3.3 Mbp, and contained more than 3,225 genes per genome.

## ANNOUNCEMENT

Brucella suis is a facultative intracellular Gram-negative bacterium belonging to the class *Alphaproteobacteria*, genus *Brucella*, which is a causative agent of brucellosis affecting wildlife, domestic animals, and humans. Five different biovars have been reported for B. suis (1). Wild boars and hares are the known reservoirs of B. suis biovar 2 in Europe (1). B. suis biovar 2 has also been shown to be the infectious agent in brucellosis developed by hunters directly exposed to wild boars (2).

Here, we report the full genomes of four B. suis biovar 2 strains isolated from free-ranging wild boars that were legally hunted in 2008 (strain WS-Be-9) and 2009 (strains WS-Be-61, WS-Be-68, and WS-Be-70) near the village of Witzwil in the canton of Bern, Switzerland. The strains were isolated from the spleens of wild boars and sent for routine parasitological and microbiological diagnostic investigations (3). Briefly, strains were cultivated on *Brucella*-selective agar (4) and identified as Brucella suis via PCR assays (5), and the biovar was typed as described elsewhere (6).

Genomic DNA was obtained by phenol-chloroform extraction and isopropanol precipitation. High-molecular-weight DNA was sheared in a Covaris g-TUBE to obtain 10- to 15-kbp fragments, which were quality assessed using the Femto Pulse system (Agilent). The purified DNA samples were barcoded with the SMRTbell Express template preparation kit v2.0 (Pacific Biosciences). Sequencing was performed using a PacBio Sequel system. Default parameters were used for all software unless otherwise specified. The assembly was performed with Flye software (release 2.6) (7), and each genome was assembled into two circularized chromosomes (chromosome 1 [chr1] and chr2). The results are summarized in Table 1.

**TABLE 1:** Summary of the four Brucella suis biovar 2 genome assemblies in this study

Strain	No. of reads	Coverage (×)	Read *N*_50_ (bp)	No. of protein-coding genes	Chromosome size (chr1/chr2) (bp)	Genome size (bp)	DNA G+C content (%)	GenBank accession no.
WS-Be-9	249,269	472	8,655	3,161	1,927,950/1,401,469	3,329,419	57.2	GCA_904066055.1
WS-Be-61	175,529	338	8,975	3,163	1,927,944/1,401,459	3,329,403	57.2	GCA_904066075.1
WS-Be-68	255,651	505	9,260	3,163	1,927,848/1,401,398	3,329,246	57.2	GCA_904066045.1
WS-Be-70	189,886	578	8,864	3,162	1,927,959/1,401,514	3,329,473	57.2	GCA_904066065.1

The draft assemblies were polished with three rounds with the software Arrow (single-molecule real-time [SMRT] Link v8 package). chr1 was rotated based on the *dnaA* gene, while chr2 was rotated using the *repC* gene. Prokka v1.13 (8) was run for annotation, with ARAGORN software (9) being used for the tRNA predictions, RNAmmer (10) for the rRNA predictions, and Prodigal (11) for the coding sequence (CDS) predictions. These four Swiss B. suis biovar 2 isolates present a chr1 with a size ranging from 1,927,848 to 1,927,959 bp and a G+C content of 57.12%, and a chr2 ranging from 1,401,398 bp to 1,401,514 bp with a G+C content of 57.33%. The WS-Be-9, WS-Be-61, WS-Be-68, and WS-Be-70 genomes contain 3,162 (±1) CDSs, 54 tRNAs, 3 rRNA operons, and 1 transfer-messenger RNA each. The functional annotation, based on BLASTp (12) analysis of the CDSs against a UniProtKB snapshot, gives an average of 889 hypothetical proteins (∼28% of the total annotated CDSs) for each genome. Complete genomes of B. suis will foster our understanding of the distribution, host restriction, and pathogenicity of the different *Brucella* species (13–16).

### Data availability.

The project has been deposited in the European Nucleotide Archive (ENA) under the identifier PRJEB39981. The PacBio reads are available under the identifiers ERR4507155 (WS-Be-9), ERR4507150 (WS-Be-61), ERR4507151 (WS-Be-68), and ERR4507152 (WS-Be-70). The genomic sequence files are registered as GCA_904066055.1 (WS-Be-9), GCA_904066075.1 (WS-Be-61), GCA_904066045.1 (WS-Be-68), and GCA_904066065.1 (WS-Be-70).
